# Effectiveness of Mass and Small Media Campaigns to Improve Cancer Awareness and Screening Rates in Asia: A Systematic Review

**DOI:** 10.1200/JGO.19.00011

**Published:** 2019-04-10

**Authors:** Désirée Schliemann, Tin Tin Su, Darishiani Paramasivam, Charlene Treanor, Maznah Dahlui, Siew Yim Loh, Michael Donnelly

**Affiliations:** ^1^ Queen’s University Belfast, Belfast, United Kingdom; ^2^ Monash University Malaysia, Bandar Sunway, Malaysia; ^3^ University of Malaya, Kuala Lumpur, Malaysia

## Abstract

**PURPOSE:**

The main objective of this systematic review was to identify whether mass and small media interventions improve knowledge and attitudes about cancer, cancer screening rates, and early detection of cancer in Asia.

**METHODS:**

The review was conducted according to a predefined protocol. Medline, EMBASE, CINAHL, Web of Science, Cochrane Library, and Google Scholar were searched in September 2017, and data extraction and rating of methodologic study quality (according to Joanna Briggs Institute rating procedures) were performed independently by reviewers.

**RESULTS:**

Twenty-two studies (reported across 24 papers) met the inclusion criteria. Most studies (n = 21) were conducted in high or upper-middle income countries; targeted breast (n = 11), cervical (n = 7), colorectal (n = 3), or oral (n = 2) cancer; and used small media either alone (n = 15) or in combination with mass media and other components (n = 5). Studies regarding cancer screening uptake were of medium to high quality and mainly reported positive outcomes for cervical cancer and mixed results for breast and colorectal cancer. The methodologic strength of research that investigated change in cancer-related knowledge and the cost effectiveness of interventions, respectively, were weak and inconclusive.

**CONCLUSION:**

Evidence indicated that small media campaigns seemed to be effective in terms of increasing screening uptake in Asia, in particular cervical cancer screening. Because of the limited number of studies in Asia, it was not possible to be certain about the effectiveness of mass media in improving screening uptake and the effectiveness of campaigns in improving cancer-related knowledge.

## INTRODUCTION

According to the Global Cancer Observatory (GLOBOCAN; April 10, 2018), Asia accounts for almost one half of newly detected cancer cases (48.4%) and more than one half of cancer deaths globally (57.3%). The most common cancers are lung, colorectal, breast, stomach, and liver cancer.^1^ Asia is a continent composed of diverse countries in terms of cultures and religions as well as economies. Most Asian countries have developing economies and are classified as low- or middle-income countries (LMICs).^2^ The strong association between the Human Development Index and age-standardized cancer incidence is reflected in the high cancer incidence rates in Asia given that most Asian countries are LMICs.^3^ LMICs experience high cancer mortality rates, and many deaths could be avoided through improved screening services that would facilitate early presentation and treatment.^4^ Population-based screening programs are lacking in most Asian countries, and the often less than optimum availability of screening facilities contributes to late detection.^4^ One of the priorities of the WHO is to reduce premature mortality from noncommunicable diseases including cancer by 25% by 2020.^5^ According to the WHO and other experts, one of the first steps towards early diagnosis is to raise awareness about cancer signs and symptoms and to encourage the seeking of help.^5^ Therefore, there is a priority need for programs that raise awareness about the warning signs and symptoms of cancer and the benefits of early detection. This form of secondary prevention should be implemented in countries in which resources for population-based screening are lacking, particularly for cancers such as colorectal and breast cancer.^6^

**CONTEXT**

**Key Objective**This research systematically reviewed studies that used mass or small media to prevent cancer in Asia.
**Key Findings** High- and middle-income Asian countries tend to focus on prevention and early detection regarding mainly breast cancer and cervical cancer. Cervical cancer small media campaigns seem to be effective in increasing screening uptake.
**Relevance** Research in low-income Asian countries is sparse due to inadequate resources. There is a need to increase empirical studies in Asia and to advance the use of research to inform and target the efficiency of prevention efforts such as public health media campaigns and plans towards reducing the significant cancer burden throughout Asia.

Evaluations of mass and small media programs in Western countries have reported promising results in terms of promoting healthy behaviors,^7^ increasing cancer-related knowledge,^8^ improving screening rates,^9,10^ and diagnosing cancer at an earlier stage.^11^ However, there is a need to identify, appraise, and summarize available evidence about the effectiveness of media campaigns to improve health-seeking behavior for cancer-related symptoms in Asia.^12^ Mass media include communication channels such as television, radio, newspapers, billboards, posters, the Internet, and smart media (ie, smartphones, smart TVs, and tablets) intended to reach large numbers of people.^7,13,14^ Small media are generally aimed at individuals rather than groups (eg, mailed letters and/or other mailed information [eg. brochures and leaflets], telephone calls, e-mails, text messages [Short Message System], and CDs or videos intended for individuals or small group viewings).^15^ The aim of this systematic review was to identify whether mass and/or small media campaigns increased knowledge and awareness about signs and symptoms of cancer, improved attitudes towards cancer screening, and increased screening attendance, self-screening, and detection rates of cancer in Asian countries.

## METHODS

This systematic review was conducted according to PRISMA guidelines and the protocol was preregistered with PROSPERO.^16^

### Search Strategy

A search strategy was developed in consultation with an information specialist with experience in devising electronic search strategies for systematic reviews. In September 2017, D.S. conducted the search, according to the predefined search terms (Appendix Table A1) and protocol, in the following databases: MEDLINE, Embase, CINAHL, Web of Science, PsycINFO, Scopus, Cochrane Library, Grey literature (ie, government reports and conference abstracts), and Google Scholar. In addition, reference lists of relevant reviews and studies were hand searched, and an individual search was conducted of relevant journals. The abstract and full-text screening of every paper was conducted by two pairs of reviewers (D.S. and M. Donnelly, T.T.S. or D.P.), and any discrepancies were resolved by a third reviewer (M. Donnelly).

### Study Selection

Publications that reported findings from campaigns using mass media (TV, radio, Internet, mobile telephone, social media, newsletters, or magazine or print advertisement), small media (brochures, leaflets, newsletters, letters, or videos), or both, were included in this systematic review if they included one of the primary outcomes under investigation: (1) cancer awareness, (2) cancer knowledge, (3) attitudes and beliefs about cancer, (4) self-efficacy to self-screen and/or see a doctor, (5) actual self-screening behavior, (6) clinical attendance because of cancer-related symptoms, (7) cancer screening attendance, and (8) numbers of cancer cases detected. Secondary outcome measures under review were the cost effectiveness of campaigns and downstaging of cancer.

#### Inclusion criteria.

Randomized and nonrandomized studies, cohort studies, quasi-experimental studies (QESs), interrupted time series, and pilot studies were eligible for inclusion if they met the following criteria: (1) were in a peer-reviewed publication, (2) were written in the English language, (3) were published before September 2017, (4) included adults 18 years of age or older, (5) were set in Asia, (6) targeted the general population or a subpopulation, (7) included mass and/or small media components that addressed at least one outcome, (8) kept individual and/or group intervention components to a minimum, and (9) investigated any cancer.

#### Exclusion criteria.

We excluded (1) interventions that were targeted at minority Asian populations (eg, Chinese living in the United States); (2) systematic reviews and cross-sectional studies, as well as conference abstracts and brief communications if sufficient details could not be obtained; and (3) studies of patients with diagnosed cancer and/or health professionals alone (studies targeting both health professionals and general populations were considered).

### Data Extraction

Heterogeneity among the studies under review did not allow for a meta-analysis to be conducted as originally planned. Instead, we systematically extracted data independently from included full-text papers into a data capture template. As with the search strategy, two pairs of reviewers (D.S. and D.P., M. Dahlui, S.Y.L. or M. Donnelly) extracted data and discrepancies between reviewers were resolved by discussion with M. Donnelly.

### Methodological Quality Assessment

We applied the relevant critical appraisal tool by the Joanna Briggs Institute (JBI) to assess the quality of each included study. Randomized controlled trials (RCTs) were scored on 13 questions and QESs were scored on nine items. D.S. and C.T. conducted the quality review, and any disagreement was resolved in discussion with M. Donnelly.

## RESULTS

**FIG 1:**
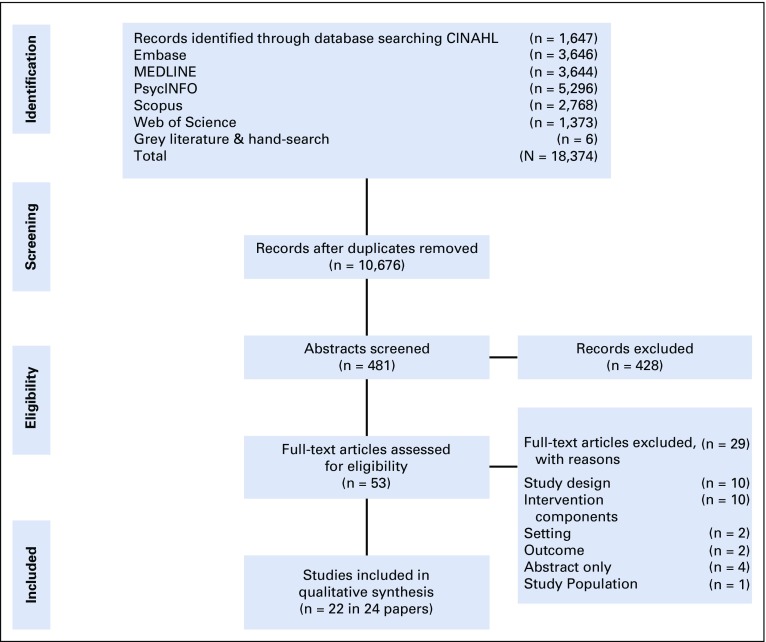
PRISMA flowchart of study selection.

The search generated 18,374 studies, of which 22 studies (published in 24 papers) met the eligibility criteria for inclusion in this systematic review (Fig 1). According to the JBI study criteria, 11 of 22 studies were RCTs (published in 13 papers) and 11 of 22 studies were QESs.

### Study Quality

RCTs were of medium to high quality (Table 1; ie, all studies met seven to 10^17,18^ JBI criteria). Criteria that were not met related mainly to blinding of participants, individuals delivering the intervention, and outcome assessors. In addition, some papers were unclear about whether random assignment had taken place or treatment allocation had been concealed. QESs were of mixed quality and ranged from meeting two of nine criteria^19^ to nine of nine criteria^20,21^ (Table 2).

**TABLE 1:**
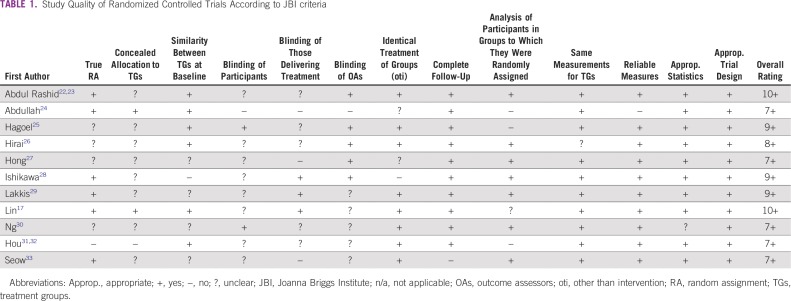
Study Quality of Randomized Controlled Trials According to JBI criteria

**TABLE 2:**
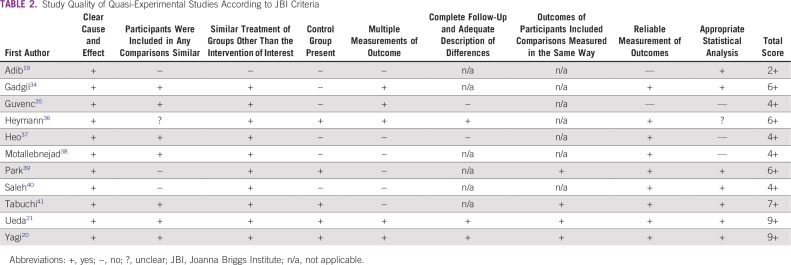
Study Quality of Quasi-Experimental Studies According to JBI Criteria

### Study Characteristics

Study characteristics are outlined in Tables 1 and 3.

**TABLE 3:**
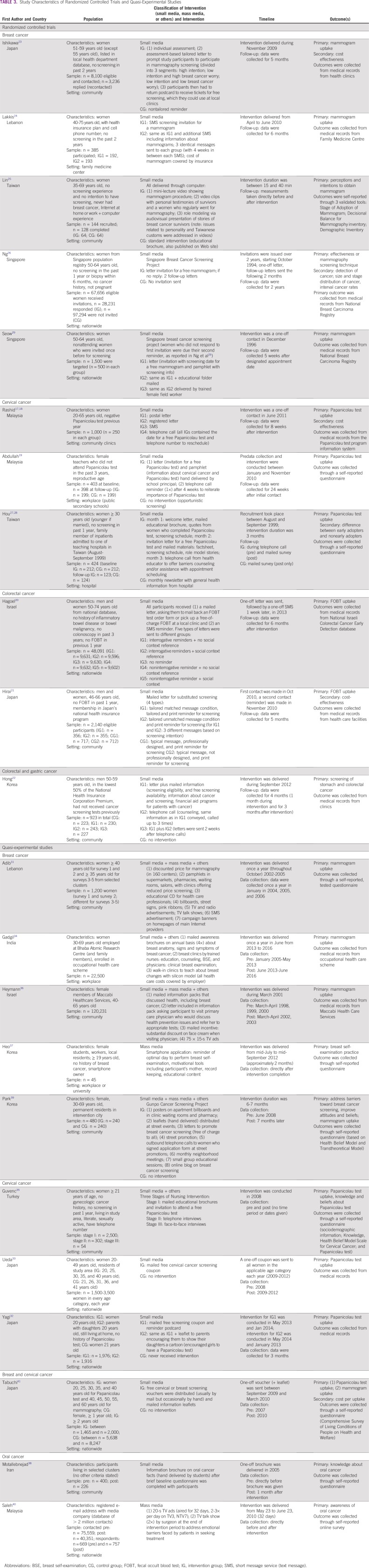
Study Characteristics of Randomized Controlled Trials and Quasi-Experimental Studies

#### Study population.

**FIG 2:**
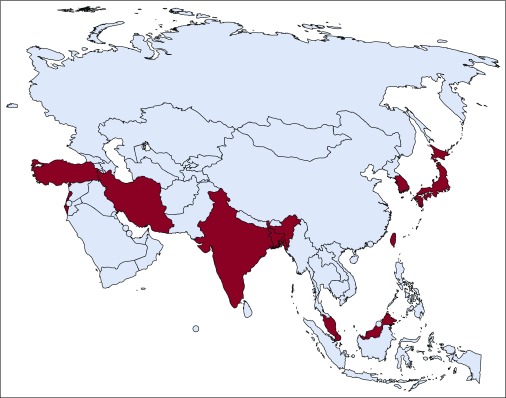
Map of Asia, highlighting countries included in interventions identified as part of this systematic review.

The majority of studies focused on breast cancer,^17,20,28-30,33,34,36,37,39,41^ followed by cervical cancer,^20-23,31,32,35,41,42^ colorectal cancer,^25-27^ oral cancer,^38,40^ and gastric cancer.^27^ The countries in which the studies were conducted included Japan,^20,21,26,28,41^ Malaysia,^22,23,40,42^ Korea,^27,37,39^ Taiwan,^17,31,32^ Israel,^25,36^ Lebanon,^19,29^ Singapore,^30,33^ India,^34^ Turkey,^35^ and Iran^38^ (Fig 2).

Individual studies targeted between 45 and 75,559 participants. Studies that aimed to increase awareness about breast and cervical cancer included women only, with the exception of two studies, one of which targeted the parents of adult daughters^20^ and another study that targeted both mothers and daughters.^37^ A study focusing on colorectal and gastric cancer targeted men only,^27^ and four studies (either targeting colorectal or oral cancer) included both men and women.^25,26,38,40^ The age range of included participants differed among studies and the type of cancer addressed (ie, cervical cancer awareness studies generally targeted women 20 years of age and older, breast cancer awareness studies targeted those 30 years of age and older, and some included women 50 years of age and older (with one exception^37^). Colorectal and gastric cancer studies included participants between 46 and 74 years of age, and oral cancer studies did not use age as an exclusion criterion. Most studies that aimed to increase screening rates included participants who did not attend screening in the past 1 to 3 years.

#### Intervention.

All RCTs of interventions used small media only (Table 3). The most common channel of communication was mailed letters, generally with the purpose of inviting participants to cancer screening. Sometimes the letters were mailed with brochures or other educational materials regarding cancer. Other small media communication channels were telephone calls and text messages (Short Message System). The RCTs included between one and four intervention groups (IGs), either comparing different channels of communication to a control group (CG) or comparing different types of messages delivered through the same channel of communication.

Included QESs used both mass and small media channels, as well as intervention components such as counseling or group education (Table 3). Two studies evaluated the impact of TV advertisements and a TV talk show,^40^ as well as a smartphone application.^37^ Three studies combined mass media (ie, TV ads, billboards, posters, street signs, radio advertisements, and a Web site) and small media communication channels, together with intervention components such as counseling, group education, discounted or free-of-charge screening, and neighborhood meetings.^19,36,39^ Four studies included small media only,^20,21,38,41^ and two studies included small media and other communication channels.^34,35^ Small media channels used in QESs included mailed letters or postcards, mailed coupons, mailed brochures or other educational materials, mailed cartoons, telephone calls, and an educational CD or video.

The few interventions that seemed to be informed by behavior change theory used constructs from the Health Belief Model,^31,32,35,39^ the Transtheoretical Model,^17,39^ and the Theory of Planned Behavior.^26,28^ One intervention was based on the Question–Behavior Effect technique,^25^ and another was developed according to the PRECEDE/PROCEED model.^39^ Few studies described the involvement of their target population in designing the intervention, although the needs assessment of the target population was described mainly in studies that used a behavior change theory.^17,35,39^

Small media studies generally targeted people in their homes, with the exception of one study that invited participants to the research center.^17^ Addresses were commonly obtained from health and population registries targeting large numbers of people (Tables 1 and 3). Other recruitment methods included convenience sampling within housing areas,^38,39^ hospitals (visiting relatives),^31,32^ or workplaces,^34,36,37^ or an e-mail list held by a mass media organization.^40^

The intervention duration and follow-up period differed among types of studies and outcomes of interest (Tables 1 and 3). Most small media interventions delivered a one-off letter or text message or followed up with a second letter, text message, or telephone call between 1 week and 3 months later and collected data on cancer screening uptake between 5 weeks and 12 months after the intervention. Other interventions posted annual brochures for up to 3 years.^34^ Small media campaigns focusing on improving cancer knowledge and perceptions conduct evaluations directly after the intervention or 1 month after.^38^ Mass media campaigns lasted from 1 month for TV only^40^ to 3 months for a smartphone application intervention only.^37^

Researchers, staff working in clinics and government screening programs, or students delivered the interventions. Trained nurses, physicians, and other clinicians undertook the screening, which was free of charge with the exception of two studies in which screening was discounted.^19,26^ Most of the studies were funded by universities and research centers.^22,23,26,33,34,37,39,42^ Other funding bodies were a pharmaceutical company,^19^ a national cancer association (nongovernmental organization [NGO]),^25^ a nursing association,^17^ a media company,^40^ a Ministry of Health (government),^20,21,26-28,30,41^ a health insurance plan,a hospital,^31,32^ and one campaign was retail-pharmacy sponsored.^36^ The funding source was unclear in two studies.^35,38^

### Study Findings

All findings are reported in Table 4.

**TABLE 4:**
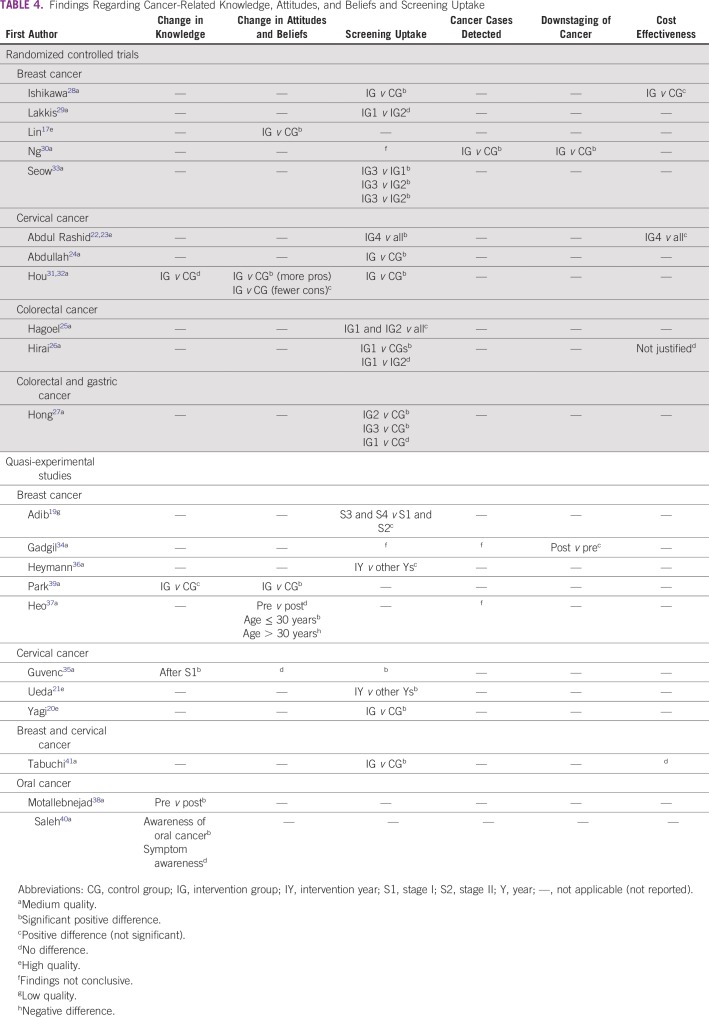
Findings Regarding Cancer-Related Knowledge, Attitudes, and Beliefs and Screening Uptake

#### Cancer-related knowledge, attitudes to cancer screening, and self-examination practice.

Change in cancer-related knowledge was assessed in one RCT and four QESs, all of medium quality.^31,32,35,38-40^ Findings from the RCT conducted by Hou et al^31^ found no between-groups difference in knowledge regarding cervical cancer and Papanicolau tests at follow-up. Conversely, Park et al^39^ found that a mixed media campaign (small and mass media plus other components) demonstrated a greater decrease in beliefs about breast cancer–related myths in Korea (nonsignificant). Furthermore, a before-and-after evaluation of a mass media campaign in Malaysia found an increase in awareness about oral cancer (ie, having heard of oral cancer), but there was no increase in knowledge about symptoms.^40^ Findings across five studies (two RCTs and three QESs) of attitudes toward screening concerning breast^17,37,39^ or cervical cancer were mixed.^31,32,35^ Studies addressing attitudes or beliefs about cancer generally described an underlying theory for the intervention design. For example, Park et al reported that a mixed media intervention based on the Transtheoretical Model, resulted in an increase in the proportion of intervention participants who progressed to the action stage (+23% in the intervention city *v* −5% in the control city) and an increase in intention to undergo mammography screening in the next 2 years (+14% in the intervention city *v* +7% in the control city).^39^ The small media intervention (combined with face-to-face interviews in stage III) that was based on the Health Belief Model did not find a change in beliefs related to cervical cancer and H tests.^35^ A small study using a smartphone application did not find a change in breast self-examination practice in general, although there was a significant increase in the number of women 30 years of age or younger conducting breast self-examination (36% to 82%, *P* = .002).^37^

#### Screening attendance, cancer diagnosis, and downstaging.

Screening uptake was the most commonly reported outcome measure (n *=* 17) for breast, cervical, and colorectal cancer. Findings from RCTs were mixed for breast (n = 4 [medium quality]) and colorectal cancer screening (n = 3 [medium quality]) and positive for cervical cancer screening (n = 3 [medium to high quality]). Only one RCT looked at gastric cancer screening.^27^ Ishikawa et al^26^ reported that a tailored letter about free breast cancer screening was significantly more effective than a nontailored reminder (odds ratio, 4.02 [95% CI, 2.67 to 6.06]; *P* < .001). Conversely, a repeated text message screening invitation combined with information about mammograms was as effective as receiving a screening invitation through text message alone.^29^ Medium-and low-quality QESs reported weak positive effects on breast cancer screening.^131,^^34,36,41^ According to one QES, breast cancer screening uptake increased over a 4-year period (not significant),^19^ and Heymann et al^36^ reported a small increase, from 3.2% to 3.8%, in another QES. High- and medium-quality QESs reported significant positive effects for cervical cancer screening,^20,21,35,41^ which were supported by high- and medium-quality RCTs.^22,24,31^ For example, Abdul Rashid et al^22^ reported a significantly greater uptake of Papanicolau tests in the IG invited by telephone compared with a mailed letter, a registered letter, or a text message (50.9%, 23.9%, 23.0%, and 32.93%, respectively; *P* < .05). Similarly, a mailed screening invitation and information followed by a telephone reminder yielded a significantly higher Papanicolau test uptake compared with no intervention (opportunistic screening; odds ratio, 2.44 [95% CI, 1.29 to 4.62]).^24^ High-quality QESs found a significant increase in Papanicolau test uptake among IG participants compared with the CG (8.7% *v* 3.6%; *P* < .001)^20^ and an increase in the first-time participation screening rate^21^ as a result of small media interventions (mailed screening coupons) in Japan. RCT participants who received a telephone call alone or a call combined with mailed information were significantly more likely to attend gastric and colorectal cancer screenings compared with the respective CGs (gastric cancer: telephone, 31.7% *v* 17.9%, *P* = .01; telephone plus post, 40.5% *v* 17.9%, *P* < .01; Colorectal cancer: telephone, 24.3% *v* 13.5%, *P* < .01; telephone plus post, 27.8% *v* 13.5%, *P* < .01).^27^

Detected cancer cases were reported in three studies. A medium-quality RCT of a small media intervention found a significant between-group difference in terms of breast cancer cases detected (IG, 4.8 of 1,000 cases *v* CG, 1.3 of 1,000 cases),^30^ whereas the interventions in two medium-quality QESs did not increase cancer case detection.^34,37^ Two medium-quality studies assessed downstaging of detected cancers as an outcome. Ng et al^30^ demonstrated a significant difference in stage of breast cancer diagnosis as a result of a small media intervention in Singapore (IG, 64% *v* CG, 26% of cases were stage 0 or 1, *P* < .001), whereas Gadgil et al^34^ reported that the proportion of smaller-sized tumors detected was higher (85.3% *v* 89.5%, *P* = .390) and the proportion of large-sized tumors detected was smaller (14.7% *v* 10.5%, *P* = .390) after the intervention. Furthermore, the proportion of cancer deaths decreased from 8.3% to 0% within 3 years from diagnosis over the study period.

#### Cost effectiveness.

Four studies reported intervention costs, with mixed findings. An intervention using assessment-based, tailored screening reminder letters to improve breast cancer screening was cost effective compared with nontailored reminders (IG, 30 USD *v* CG, 52 USD),^28^ whereas a tailored message condition was not more cost effective than an unmatched message condition for colorectal cancer screening.^26^ Abdul Rashid et al^23^ compared different small media campaigns to increase cervical cancer screening and found that a telephone call was the most cost-effective method. An intervention that paid out-of-pocket costs for breast and cervical cancer screenings in Japan improved cancer screening uptake, although the intervention was not cost saving because of the high cost of screening.^41^

## DISCUSSION

Findings from this systematic review suggest that small media interventions (eg, interventions using mailed materials, text messages, and telephone calls) may be effective in improving screening uptake for breast, cervical, colorectal, and gastric cancer in Asian countries. The number of studies using mass media channels was too small to draw conclusions about their effectiveness. There was also insufficient evidence to indicate that small or mass media campaigns improved knowledge or attitudes toward cancer. The lack of mass media campaigns is likely to be related to (1) the high costs involved in running campaigns using TV and radio advertisements and (2) the lack of campaign evaluation of campaigns run by the government and NGOs. The only nationwide mass media campaigns included here received funding from media channels for TV advertisements.

The findings regarding screening were mainly from studies conducted in high or higher middle-income countries (Japan, South Korea, Taiwan, Singapore, Malaysia, Israel, Turkey, Lebanon, and Iran). The absence of studies in low and lower middle-income countries may be explained by a lack of resources to conduct screening programs, as well as a lack of screening facilities. Most studies reported a one-off follow-up, and only a few studies evaluated the impact of such programs in the long term. Studies from Western countries suggest that screening programs have to be run repeatedly to maintain uptake over time.^43^

Surprisingly, the two most common cancers in Asia, lung and liver cancer, were not addressed by any study in the systematic review. The majority of lung and liver cancer programs tend to focus on prevention (ie, smoking cessation and hepatitis B vaccination) instead of symptom education and early detection. However, the high number of lung and liver cancer cases suggests that there is a need for early detection and awareness programs to supplement prevention programs and to detect and treat these cancers early. The under-researched number of cancer cases detected and downstaging of cancer may be related to the poor quality or absence of adequate data collection systems in LMICs. Bhoo-Pathy et al^44^ reported that only one in three Asian countries collected data on cancer incidence, and only one in six countries monitored cancer mortality. In turn, inadequate or absent routine data collection is likely to hinder cost-effectiveness analysis of interventions.

Eight studies (40%) reported implementation issues. Findings highlighted that between 21.2% and 34.4% of letters, mailed brochures, or text messages were never received because of incorrect addresses or telephone numbers^22,29^ and that approximately 43.5% of targeted participants never read the brochure they received.^38^ One study using mass and small media highlighted that 50% of participants reported that they had heard about the campaign.^19^ Reasons why women refused free cervical cancer screening after the first contact included no time and embarrassment during screening.^35^

Findings presented in this systematic review are in line with the findings of two systematic reviews focused mainly on Western countries.^15,43^ Furthermore, Hou et al^12^ concluded that small media were effective in improving screening uptake among Asians (including Asians living abroad). To the best of our knowledge, the systematic review presented in this article is the first review focusing on Asians living in Asia and takes account of the different health care systems and resources in Asian countries compared with Western countries. In addition, the review extracted information about small and mass media campaigns specifically, rather than educational interventions in general; these data will be informative for the design and development of early detection cancer programs that plan to use this mode of delivery.

To the best of our knowledge, this systematic review delivers the best available up-to-date reliable evidence about small and mass media cancer screening interventions in Asia. Most studies in this systematic review were deemed to be of medium quality according to the results of the application of the JBI methodologic checklists. However, a consideration of individual studies in the context of the target interventions might suggest that some may be higher in methodologic quality. For example, the scoring of criteria such as blinding may not be realistic for these types of population-based educational interventions.

Often, data collected from medical records or cancer registries in LMICs are not complete or reliable because of a lack of resources. For example, the cancer registry in Malaysia relies on voluntarily supplied information,^45^ and because of the dual-tiered health care system, evidence from private clinics and hospitals is often lacking. Many interventions and campaigns run by governments and NGOs in LMICs are evaluated internally and are not published in scientific journals and, therefore, may be missed.

Few of the studies included offered minimal contact with participants (eg, neighborhood meetings, telephone contact, and so forth) and we do not know the extent to which this personal contact is important for intervention success. Due to the limited number of studies, no conclusions can be drawn about whether interventions that applied a theory were more effective than atheoretical studies or whether there are differences in effectiveness between screening tests. However, a recent systematic review by Senore et al^43^ suggested that different colorectal cancer screening methods yielded different results regarding screening uptake.

Because some studies compared one intervention with another intervention (eg, tailored messages *v* nontailored messages), no conclusions can be drawn from some interventions regarding the effectiveness of the intervention compared with no intervention. Our review covered a limited number of high and higher middle-income countries, and findings may not be applicable to other LMICs in Asia (Fig 2). Furthermore, few studies looked at using different methods to target different age groups. However, it was suggested that younger women may be better disposed to smartphone applications^37^ as well as to being influenced by their parents.

Mailed information and an invitation for a free screening, as well as mailed information combined with a telephone reminder, seem to be effective in increasing screening uptake. High-quality studies in this review may serve as important resources to inform screening interventions in Asian countries. A limited number of interventions in this systematic review evaluated screening programs over an extended time period, and future studies should investigate screening engagement in the long term.^43^

Few studies addressed knowledge and attitudes regarding cancer and cancer screening. However, in some LMICs, lack of knowledge, misbeliefs, negative attitudes toward cancer treatment, and distrust in Western medicine are still significant barriers toward screening,^46,47^ and these barriers must be addressed to improve screening uptake in Asia. Understanding barriers toward screening in the target population is a key research goal,^43^ and basing interventions on theoretical components may improve effectiveness. The two most commonly applied theories in cancer education programs in Asia are the Transtheoretical Model and the Health Belief Model.^12^

Mass media campaigns are run yearly by NGOs and industry,^48^ but they do not seem to be subject to rigorous evaluation. To identify whether mass media are cost effective and worthwhile to be used by policy makers and public health practitioners for public education in Asia, there would be considerable merit in NGOs and campaigning bodies exploring collaboration with academicians with a view to rigorously evaluating public health improvement programs.

Findings from this systematic review suggest that small media cancer awareness–raising campaigns are effective in increasing cancer screening rates for breast and cervical cancer, and limited evidence is available for colorectal cancer. Evaluation of mass media campaigns is required to improve understanding about the importance (or otherwise) of these campaigns in public health education. Additional research is needed to assess the cost effectiveness of media interventions for cancer screening in Asia.

## Figures and Tables

**TABLE A1:**
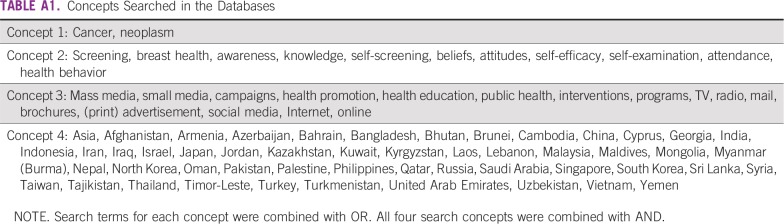
Concepts Searched in the Databases
